# Major issues and their solutions in the management of HIV and TB — from the laboratory to the patient and population

**DOI:** 10.1186/1758-2652-13-S4-O23

**Published:** 2010-11-08

**Authors:** M Schechter

**Affiliations:** 1 AIDS Research Laboratory, Hospital Universitario Clementino Fraga Filho, Universidade Federal de Rio de Janeiro, Rio de Janeiro, Brazil

## 

HIV is the strongest known risk factor for developing active tuberculosis. In individuals with Mycobacterium tuberculosis infection, the risk of developing active disease is 20 or more times greater in persons living with HIV than HIV negatives. Tuberculosis is the most common HIV-associated illness in resource poor settings and the most common opportunistic infection in patients receiving HAART in developed countries. Of the 33 million people estimated to be living with HIV globally in 2008, approximately 1.5 million were also tuberculosis patients. In that same year, tuberculosis was responsible for 25% of all deaths in HIV-infected individuals. Given the synergistic consequences of the two epidemics, has recommended several collaborative activities as part of core HIV and TB prevention, care and treatment services. In this conference, some of the most important recent advances in prevention, diagnosis, and treatment will be critically reviewed.

